# Supplementary material for: A two-component system signaling hub controls enterococcal membrane remodeling in response to daptomycin
Source: Proc Natl Acad Sci U S A. 2026 Apr 21;123(17):e2532437123. doi: 10.1073/pnas.2532437123 (PMC13123819; doi:10.1073/pnas.2532437123)
Supplement: Supplementary file 1 — Appendix 01 (PDF) [file pnas.2532437123.sapp.pdf]

PNAS

Supporting Information for

A two-component system signaling hub controls enterococcal membrane remodeling in response to daptomycin

Cristina Colomer-Winter[1], Zeus J. Nair[2], Jerome Y. J. Chua[3], Soukayna Jabli[1], Mélanie Roch[1], Amaury Cazenave-Gassiot[4,5], Roberto Sierra[1,6], Diego O. Andrey[1,6,7], Shu Sin Chng[3,8] and Kimberly A. Kline[1,2,3]*.

*Corresponding author: Kimberly A. Kline
Email: Kimberly.Kline@unige.ch

This PDF file includes:

Supporting text
Figures S1 to S4
Tables S1 to S4
Legends for Datasets S1 to S2
SI References

Other supporting materials for this manuscript include the following:

Datasets S1 to S2

Supporting Information Text

Extended methods

Growth curves in the presence of DAP

Growth of *E. faecalis* wild-type in the presence of DAP was examined to determine a sub-inhibitory concentration for downstream experiments. Overnight cultures of single colonies were prepared in BHI supplemented with 50 mg/L $CaCl_2$ (from now on referred to as BHIc). The next day, overnight cultures were diluted 1:40 in fresh BHIc medium and incubated to early exponential phase at 37°C. Then, cultures were diluted 1:100 into BHIc containing increasing concentrations of DAP in a 96-well plate and growth was monitored by measuring OD_{600} at regular timepoints for up to 24h using a Tecan plate reader. Three biological replicates were monitored in parallel for each DAP concentration. To assess the effect of DAP on exponentially growing cultures, the protocol was adapted with minor modifications. Two different set-ups were tested. To monitor growth over 24 hours upon DAP exposure, overnight cultures were diluted 1:40 into fresh BHIc in a 96-well plate and growth at 37°C was monitored until the cultures reached exponential phase. Then, increasing concentrations of DAP were immediately added to corresponding wells and growth monitoring was resumed for up to 24h. To determine OD_{600} and CFU values after 1 hour treatment, overnight cultures were diluted 1:40 into 15 mL fresh BHIc in a 50 mL Falcon tube and incubated to early exponential phase (~ 0.25) at 37°C. Then, cultures were immediately split into three 15 mL tubes with 4 mL culture each and treated with either 0 µg/mL, 2 µg/mL, or 8 µg/mL DAP. An aliquot was removed for OD_{600} reading and CFU enumeration on BHI plates, and tubes were immediately incubated at 37°C for 1 hour. After 1 hour, OD_{600} and CFU enumeration was repeated. BHI plates were incubated overnight at 37°C.

Liquid chromatography tandem mass spectrometry (LC-MS/MS) analysis of lipids

Overnight cultures of *E. faecalis* were sub-cultured 1:100 into fresh BHI medium and grown to early exponential phase (OD_{600}~0.25). Then, cultures were split into two tubes and one of them was treated with 2 µg/mL DAP for 1 hour at 37°C. Cultures were pelleted and washed with 1 mL phosphate buffered saline (PBS) before proceeding with lyophilization.

Lipids were extracted from lyophilized cell pellets using a modified Bligh and Dyer method, where the extraction solvent contained chloroform/methanol in a ratio of 1:2 (vol/vol) as previously described (1, 2). PG (PG 14:0) and LPG (LPG 16:0) were prepared as 0.1 mg/mL stocks in the extraction solvent and 45 µL of PG 14:0 and 36 µL of Lys-PG 16:0 were added to the lyophilized samples to serve as internal standards. Additional extraction solvent was then added to a final volume of 900 µL and lipid extraction was carried out as described previously (1). The dried lipid extract was resuspended in 30 µL chloroform/methanol (1:1 vol/vol) and stored at −80°C until the mass spectrometry analysis was performed.

PG, LPG and DGDAG were quantified by LC-MS/MS using multiple reaction monitoring (MRM) using previously described methodologies (1, 2). An Agilent 6490 QqQ mass spectrometer connected to a 1290 series chromatographic system was used with a Kinetex 2.6 µm HILIC column (100 Å, 150 × 2.1 mm) (Phenomenex, USA). Electrospray ionization (ESI) was used to ionize lipids. Each lipid molecular species was analysed using a targeted MRM approach containing transitions for known precursor/product mass-to-charge ratio (m1/m3). For PG and LPG measurements, signal intensities were normalized to the spiked internal standards to obtain relative measurements and further normalized against the initial lyophilized cell pellet weight (1, 2). Due to the lack of commercially available standards for DGDAG and MGDAG, normalization to standards was not performed for DGDAG. Instead, signal intensities were normalized to the weight of the lyophilized cell pellets for semi-quantitative measurements. For data visualization purposes, log_2-fold changes between treated and untreated samples were calculated and plotted as heatmaps. Fold-change in LC-MS/MS lipid species quantification (nM/mg dry weight or intensity/mg dry weight) between DAP-treated vs untreated *E. faecalis* WT samples was calculated for each lipid species and individual bioreplicate. Fold-change was log_2 transformed and the average of 3 to 5 bioreplicates was calculated. The heat-map represents the average increase or decrease in lipid species when *E. faecalis* is treated with 2 µg/mL DAP for 1 hour compared to untreated. Since not all fatty acid tail lengths were detected in PG, LPG, and DGDAG, only the fatty acid tails that are common to all three lipid classes were plotted.

RNA-Sequencing

Overnight cultures were sub-cultured 100-fold into fresh BHI supplemented with 50 mg/L $CaCl_2$ and grown at 37°C under static conditions to mid-log phase (OD_{600} ~0.5). Then, 2 µg/mL DAP was added to the culture and incubated for 15 min. Control cultures were left untreated. For RNA extraction, cultures were harvested and treated with RNAprotect (Qiagen, USA) to stabilize RNA. Then, cells were lysed with 20 mg/mL lysozyme (Sigma-Aldrich, USA) in lysis buffer (10 mM Tris-HCl at pH 8, 50 mM NaCl, 1 mM EDTA, 0.75 M Sucrose) at 37°C for 1 h. Following centrifugation, the pellet was resuspended in 1 mL of TRIzol and 200 µL of chloroform, and incubated at 4°C for 2 min. After centrifuging at 12,000g at 4°C for 15 min, the top aqueous phase was mixed thoroughly with an equal volume of 80% ethanol. Total RNA was then isolated using the RNeasy Mini Kit (Qiagen, USA) according to the manufacturer's recommendations. DNA was depleted via on-column DNase I treatment (Qiagen, USA). RNA quality (RINe ≥8) was assessed using the RNA ScreenTape system (Agilent Technologies, USA), while DNA contamination (≤10%) was quantified using the Qubit RNA BR and dsDNA HS Assay kits (Invitrogen, USA). cDNA library preparation and ribosomal RNA depletion was performed by the in-house sequencing facility using the Illumina Total RNA Prep with Ribo-Zero Plus kit (Illumina, USA). Paired-end sequencing (150x150bp) was performed on the Illumina HiSeqX v2.5 system. The quality of the sequencing data was assessed

using FastQC (version 0.11.9)(3). Adapter and quality trimming were performed using bbduk (BBMap version 38.96)(4). The trimmed reads were mapped to the *E. faecalis* OG1RF reference genome (GenBank accension CP002621.1) using the BWA-MEM algorithm of Burrows-Wheeler Aligner (version 0.7.17-r1198-dirty)(5) with the default settings. Transcript abundance was determined using htseq-count (HTSeq version 2.0.1) (3) with intersection-strict mode. Differential gene expression (DGE) analysis was performed in R (version 4.3.1) using edgeR (version 3.42.4)(6).

Radiolabelled pulse-chase lipid thin-layer chromatography (TLC)

Radiolabeling of lipids was performed as previously described with the following modifications (2, 7). For labelling of total lipids, overnight cultures of *E. faecalis* grown in BHI supplemented with 50 mg/L $CaCl_2$ were diluted 1:100 in fresh media spiked with 0.5 µCi/mL [^{14}C]-acetate (Perkin Elmer) and grown to OD_{600} of 0.25. Cultures were then split into two tubes and one of them was treated with 2 µg/mL DAP for 1 hour. For labelling of newly synthesized lipids during DAP exposure, overnight cultures of *E. faecalis* grown in $CaCl_2$-supplemented BHI were diluted 1:100 in fresh media and grown to OD_{600} of 0.25. Cultures were then spiked with 0.5 µCi/mL [^{14}C]-acetate and treated with 2 µg/mL DAP for 1 hour. For labelling of pre-existing lipids prior to DAP exposure, overnight cultures of *E. faecalis* grown in $CaCl_2$-supplemented BHI were diluted 1:100 in fresh media spiked with 0.5 µCi/mL [^{14}C]-acetate and grown to OD_{600} of 0.25. Cultures were then pelleted, washed with 1 mL of fresh media, and resuspended in fresh media supplemented with 8.5 mM of unlabelled sodium acetate (Sigma-Aldrich, USA). Cultures were then treated with 2 µg/mL DAP for 1 hour.

For all three setups, after incubation with DAP, lipids were extracted from these cultures as previously described and resuspended in 50 µL of chloroform-methanol solution (1:1 vol/vol) (1). Radioactive counts of the lipid extracts were measured, and the lipid extracts spotted on to silica gel-coated thin-layer chromatography (TLC) plates (Merck, USA) while normalizing to scintillation counts as previously described (2). TLC plates were developed in preequilibrated TLC chambers with a chloroform:methanol:water (65:25:4) solvent system. TLC plates were then visualized by exposure to a storage phosphor screen (GE health care, USA) overnight, and read using a Storm Phosphorimager (GE health care, USA). Percent intensity of each spot was calculated relative to total intensity. For data visualization purposes, log_2-fold changes between treated and untreated samples were calculated and plotted as a heatmap. Values were calculated for newly synthesized lipids and for pre-existing lipids separately. Fold-change in percent intensity of lipid TLC spots between DAP-treated vs untreated *E. faecalis* WT samples was calculated for each lipid spot and individual bioreplicate. Fold-change was log_2 transformed and the average of 3 bioreplicates was calculated. The heat-map represents the average increase or decrease in either newly synthesized

or recycled lipid classes when *E. faecalis* is treated with 2 µg/mL DAP for 1 hour compared to untreated.

Construction of deletion strains

Deletion of *ltaS1*, *ltaS2,* and *sapRS* in the *E. faecalis* OG1RF background was carried out using the pGCP213 temperature-sensitive Gram-positive plasmid as previously described (8). Construction of Δ*bsrRS* was carried out using the CRISPR-Cas12a system as previously described for *E. faecium* (9). Strains, plasmids, primers and antibiotics used for cloning are described in **Tables S1, S2, and S3**. All gene deletions were confirmed by PCR sequencing of flanking sequences in mutant strains.

Briefly, ~ 0.5 kb PCR products flanking the *sapRS, ltaS1,* or *ltaS2* coding sequences were amplified with the primers listed in (**Table S3**). Restriction enzyme sites were created as silent mutations whenever possible to aid in cloning. The amplicons included the first and last ~15 amino acid codons of the coding DNA to avoid unanticipated polar effects. The flanking regions were fused together by overlap extension PCR to generate the final insert. Then, insert and pGCP213 were digested with appropriate restriction enzymes and ligated with T4 ligase following the manufacturer's instructions. The generated plasmids were transformed into competent *E. coli* Stellar cells, transformants were screened with M13 primers and verified by PCR and by Sanger sequencing. Electroporation into electrocompetent *E. faecalis* OG1RF and final isolation of mutant strains were carried out as previously described (10). Colonies were screened with the respective screening primers to identify clones harboring the expected gene deletions. Double mutants were obtained electroporating the different pGCP213 constructs into electrocompetent Δ*ltaS2* or Δ*liaFSR* single mutants. A triple mutant was obtained by electroporating the pGCP-*ltaS1* plasmid into the Δ*liaFSR*Δ*sapRS* double mutant strain.

For construction of Δ*bsrRS*, a 1.3 kb synthetic insert was designed, composed of: (i) the Cas12a target sequence, located immediately downstream of a TTTV protospacer adjacent motif (PAM) within the gene of interest, (ii) ~500 bp sequences homologous to the regions upstream and downstream of the target gene for recombination. The sequence of the synthetic insert is provided in **Table S3**. The synthetic insert was amplified using primers insert-F and insert-R and subsequently cloned into the plasmid pJC005, which had been linearized by PCR with the primers pJC005-inv-1 and pJC005-inv-2, using *in vivo* assembly in *E. coli* Stellar competent cells (11, 12). The resulting recombinant plasmid was extracted, verified by Sanger sequencing (Microsynth), and electroporated into competent *E. faecalis* OG1RF prepared by overnight growth in BHI 7% glycine, 0.5M sucrose. Transformants were selected on Brain Heart Infusion (BHI) agar supplemented with 50 µg/mL erythromycin. Cas12a expression was induced with 250 ng/mL anhydrotetracycline (ahTC) in the presence of 50 µg/mL erythromycin. Successful gene knockout was confirmed by

PCR using primers screen-F and screen-R. Plasmid curing was carried out by two successive passages in BHI broth containing ahTC but lacking erythromycin.

Whole genome sequencing

Oxford Nanopore Technologies was used to perform whole genome sequencing on parent OG1RF and deletion strains Δ*ltaS1*, Δ*ltaS2*, and Δ*ltaS1*Δ*ltaS2* and confirm absence of suppressor mutations. Genomic DNA was extracted from *E. faecalis* WT and mutants using the Wizard HMW DNA Extraction Kit (Promega) following the manufacturer's instructions. Libraries were prepared using the Rapid Barcoding Kit SQK-RBK114.24 and sequenced in R10.4.1 flow cell on a GridION instrument. Reads were basecalled with Dorado (v.0.9.2) in super accuracy mode, genomes were assembled with flye (v.2.9.1) and polished with medaka (v.2.0.1). To refine SNPs and indel detection reads, knockout strains were directly compared with WT reads and with the *E. faecalis* OG1RF reference genome (NCBI assembly accession: GCA_000172575.2) using the medaka pipeline available at https://github.com/BioinfoSupport/nanopore_compare. All putative single-nucleotide polymorphisms (SNPs) and insertions/deletions (indels) flagged by the pipeline were manually verified by read visualization using the Integrative Genomics Viewer (IGV) (13).

Figures

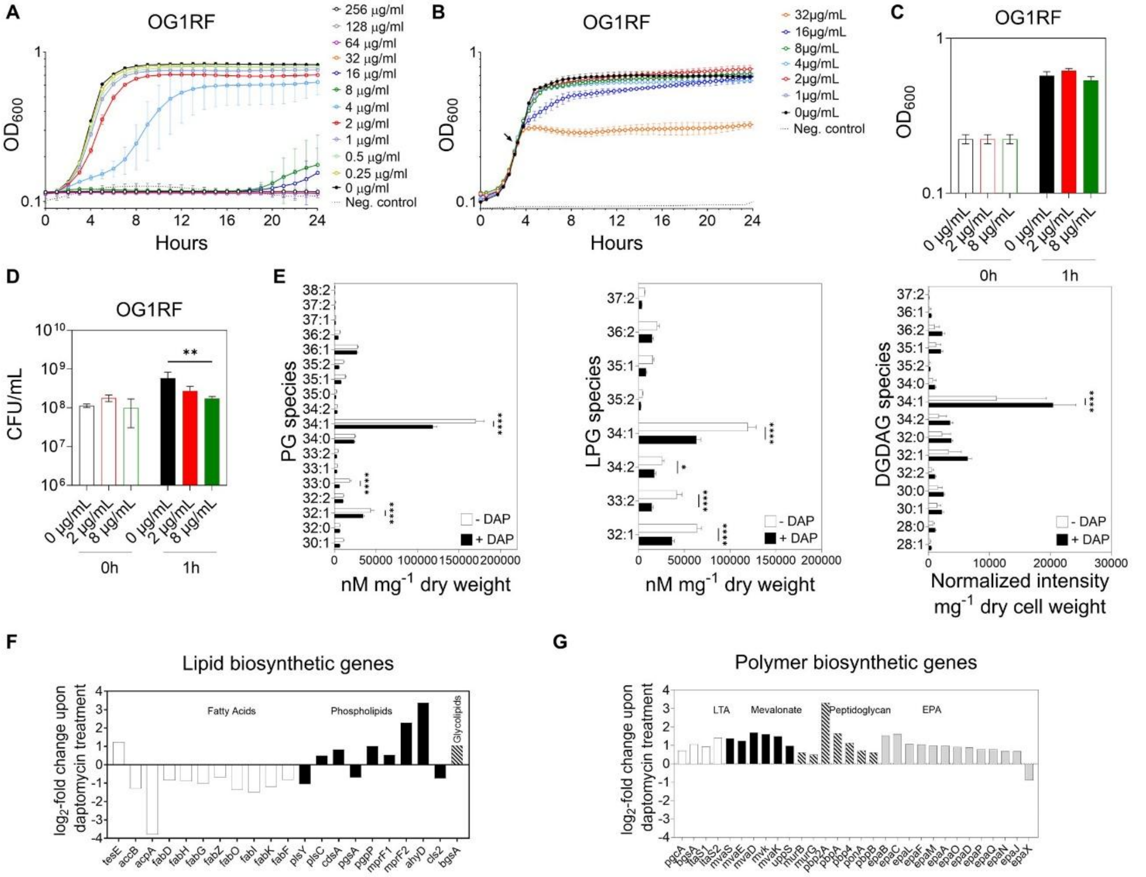

Fig. S1. **(A, B)** Growth of WT *E. faecalis* OG1RF in the presence of DAP. (A) Exponentially growing cells were diluted 1:100 into fresh BHI supplemented with $CaCl_2$ and increasing concentrations of DAP. (B) Overnight cultures were diluted 1:100 into fresh BHI supplemented with $CaCl_2$ and incubated at 37°C until exponential growth phase. Then, cells were immediately challenged with increasing concentrations of DAP after which incubation and absorbance monitoring was resumed. The timepoint at which DAP was added to the cultures is indicated by an arrow. In both setups, growth was monitored by measuring OD_{600} (A) every hour or (B) every 30 min for up to 24h using a Tecan plate reader. The graph shows the average and SD of three biological replicates. **(C, D)** Absorbance and CFU enumeration of OG1RF WT cultures treated with DAP for 1 hour. OG1RF WT was grown to early exponential phase and treated with either 0 μg/mL, 2 μg/mL, or 8 μg/mL DAP. (C) Absorbance (OD_{600}) and (D) CFU/mL were determined upon DAP addition (0h) and after 1 hour incubation at 37°C. Differences in absorbance (OD_{600}) and in log_{10} CFU between the three

conditions were analysed via a two-way ANOVA followed by Dunnett's multiple comparison test (** $P \leq 0.01$) **(E)** LC-MS/MS quantification of individual PG, LPG, and DGDAG species in treated and untreated WT *E. faecalis* OG1RF. Carbon length and saturation status of fatty acids are shown in the y-axis. Each bar represents the mean ± standard error of 3 to 5 biological replicates. Differences in lipid content were analysed via a two-way ANOVA followed by Šídák's post-test (* $P \leq 0.05$, **** $P \leq 0.0001$). **(F, G)** Transcriptional expression of selected (F) lipid and (G) cell envelope-associated polymer biosynthetic genes during DAP treatment. *E. faecalis* OG1RF was treated with 2µg/mL DAP for 15 min prior to RNA extraction and downstream RNA-Seq analysis. Bar graphs show the mean $\log_2$ fold-change of three biological replicates comparing treated to untreated OG1RF cultures. All values shown were statistically significant (FDR < 0.05). LTA = Lipoteichoic Acid, EPA = Enterococcal Polysaccharide Antigen.

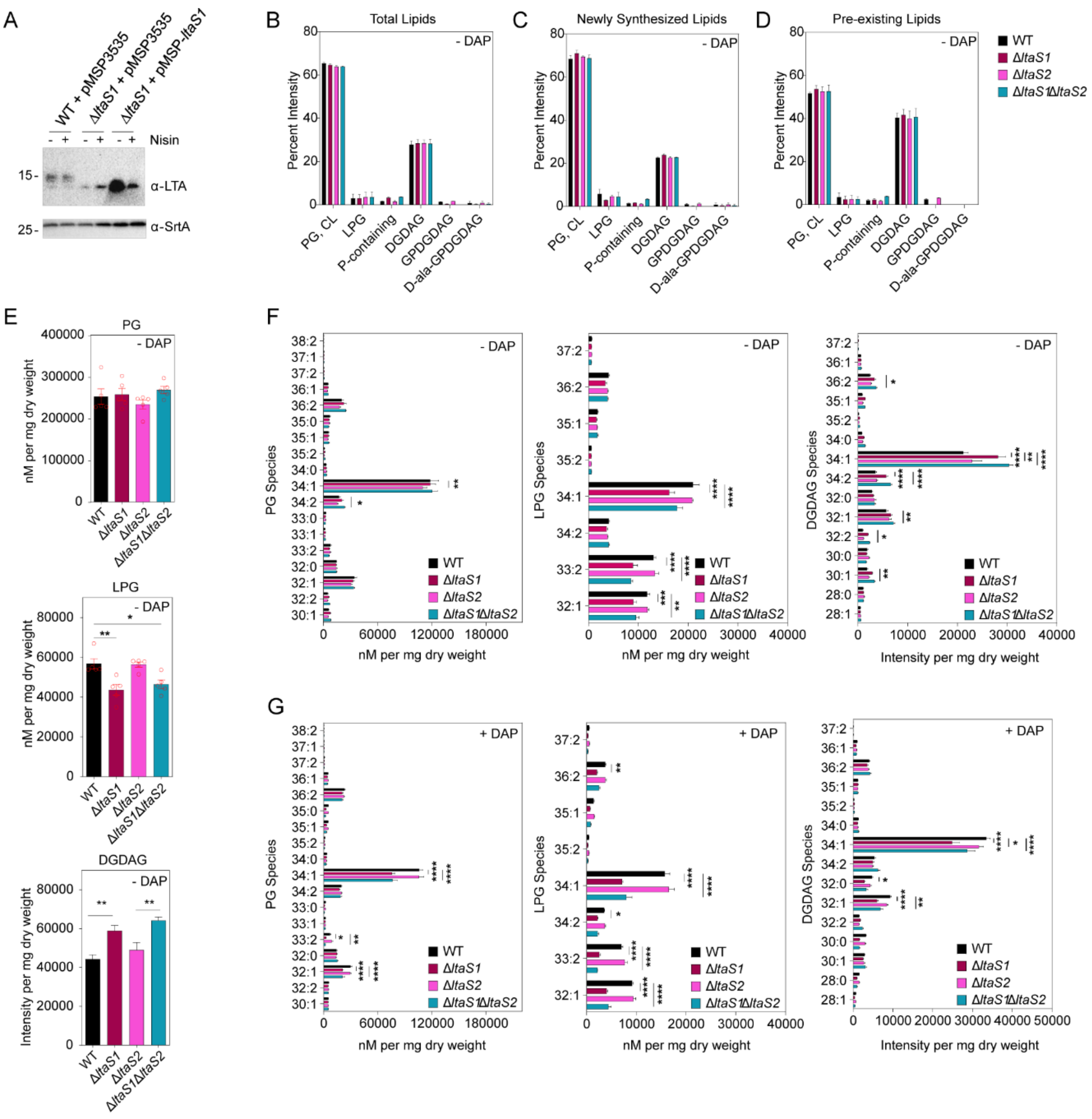

Fig. S2. **(A)** LTA Western Blot of cell extracts of OG1RF WT and Δ*ltaS1* carrying the empty pMSP3535 plasmid or Δ*ltaS1* carrying the pMSP-*ltaS1* vector. Overnight cultures were incubated with or without 25 ng/mL nisin prior to cell lysis to determine LTA production under induced and uninduced conditions, respectively. SrtA was used as a loading control. Size is denoted in kDa. Of note, the nisin-inducible promoter was reported to be leaky (14). **(B, C, D)** Densitometric analysis of [14C] radiolabeled 1D TLC spots of (B) total lipids, (C) newly synthesized lipids, and (D) pre-existing lipids in parent and LTA-defective strains in the absence of DAP (-DAP). Each bar represents the mean ± standard error of measurement calculated from 2 biological replicates. Spot identities were determined using lipid standards. Results were analyzed via two-way ANOVA. Differences were not statistically significant. **(E)** LC-MS/MS quantification of total PG, LPG, and

DGDAG content of untreated WT and LTA-deficient *E. faecalis* cells. PG and LPG were normalized to internal standard and cell pellet weight, while DGDAG intensity was normalized to cell pellet weight for a semi-quantitative measurement. Each bar represents the mean ± standard error of 3 to 5 biological replicates. Differences in lipid content were analysed via one-way ANOVA. (* $P \leq 0.05$, ** $P \leq 0.01$). **(F, G)** LC-MS/MS quantification of PG, DGDAG, and LPG lipid species of (F) untreated (- DAP) and (G) treated (+ DAP) *E. faecalis* WT and LTA-deficient cells. PG and LPG were normalized to internal standard and cell pellet weight, while DGDAG intensity was normalized to cell pellet weight for a semi-quantitative measurement. Each bar represents the mean ± standard error of 3 to 5 biological replicates. Differences in lipid content were analysed via one-way ANOVA. (* $P \leq 0.05$, ** $P \leq 0.01$, *** $P \leq 0.001$, **** $P \leq 0.0001$).

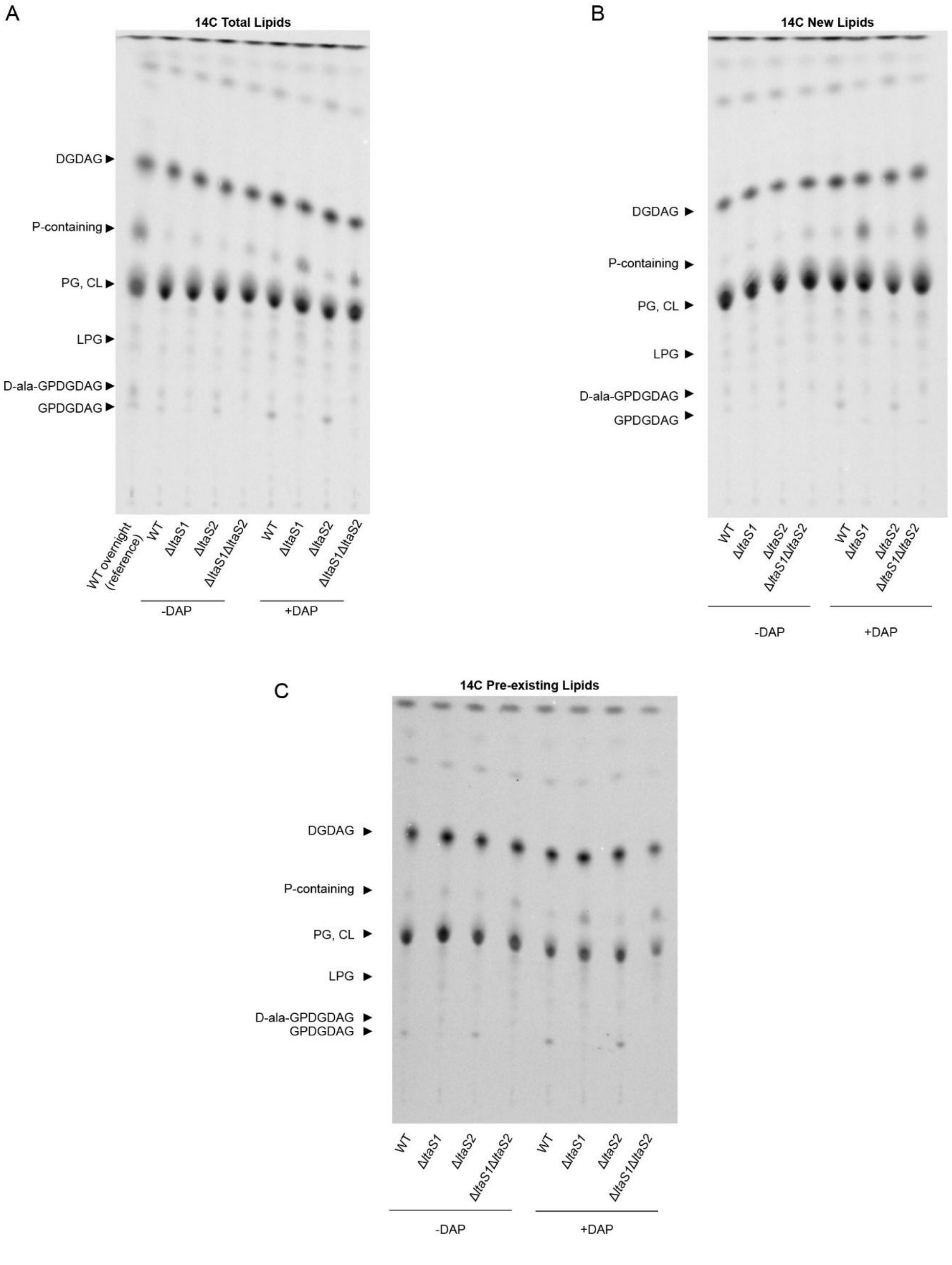

Fig.S3. Representative 14C radiolabeled 1D-TLC of lipid extracts using chloroform:methanol:water (65:25:4). **(A)** Total lipids from exponentially growing WT, Δ*ltaS1*, Δ*ltaS2,* and Δ*ltaS1*Δ*ltaS2* cells treated with 2µg/mL DAP for 1 hour. Untreated cultures served as control. Lipids were radiolabelled

with 14C-acetate immediately after overnight subculture. Standards and an overnight WT culture were used to aid in spot identification as previously reported (2). **(B)** New lipids synthesized from new fatty acids and incorporated into the cell membrane upon DAP treatment. Radiolabelled 14C-acetate and DAP were supplemented simultaneously and incubated with cells for 1 hour at 37°C. DAP-treated and untreated controls of WT, ∆*ltaS1*, ∆*ltaS2,* and ∆*ltaS1*∆*ltaS2* cells are shown. **(C)** Pre-existing lipids that were already present in the cell membrane before DAP treatment. Radiolabelled 14C-acetate was supplemented immediately after overnight culture. Before DAP treatment, radiolabelled acetate was replaced with cold acetate. Then, DAP was added and cells were incubated for 1 hour at 37°C. DAP-treated and untreated controls of WT, ∆*ltaS1*, ∆*ltaS2,* and ∆*ltaS1*∆*ltaS2* cells are shown.

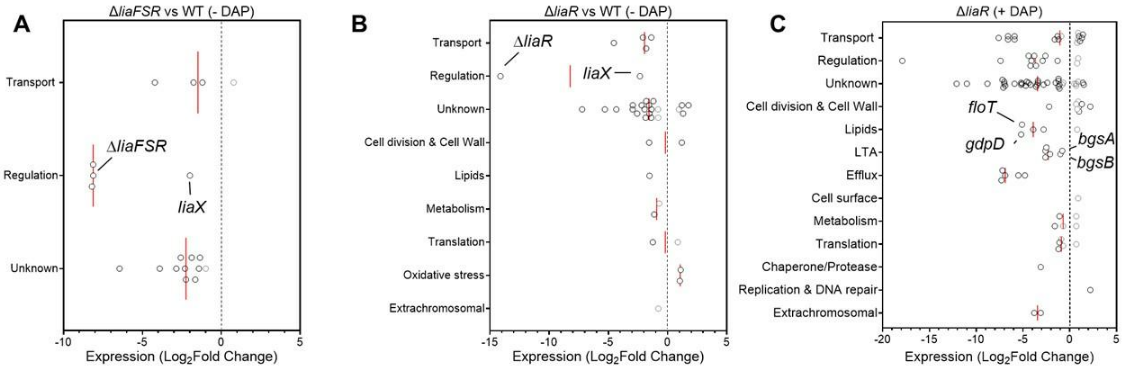

Fig. S4. **(A-C)** RNA-Seq of (A) untreated OG1RF Δ*liaFSR*, (B) untreated OG1RF Δ*liaR*, and (C) DAP-treated OG1RF Δ*liaR* compared to their respective WT controls. Exponentially grown cells were either treated with 2µg/mL DAP or left untreated for 15 minutes prior to RNA extraction. Analysis was performed on 3 biological replicates per condition. The graph shows the functional classification of differentially expressed genes. Genes exhibiting $Log_2FC \geq 1.0$ with $FDR \leq 0.05$ are represented with black circles, while genes exhibiting $Log_2FC \leq 1.0$ with $FDR \leq 0.05$ are represented with grey circles; selected genes are labelled for ease of identification.

Tables

Table S1. Bacterial strains and plasmids

Strain	Description[a]	Reference
<u>*E. faecalis*</u>		
OG1RF	Reference sequenced strain; Fus^R and Rif^R	(15, 16)
Δ*ltaS1*	OG1RF *ltaS1* deletion	This work
Δ*ltaS2*	OG1RF *ltaS2* deletion	This work
Δ*ltaS1*Δ*ltaS2*	OG1RF *ltaS1* and *ltaS2* deletion	This work
OG1RF + pMSP3535	Parent strain carrying the empty pMSP3535 plasmid	This work
Δ*ltaS1* + pMSP3535	Δ*ltaS1* strain carrying the empty pMSP3535 plasmid	This work
Δ*ltaS1* + pMSP-*ltaS1*	Δ*ltaS1* strain carrying the pMSP-*ltaS1* construct	This work
Δ*liaFSR*	OG1RF *liaFSR* deletion	(8)
Δ*liaR*	OG1RF *liaR* deletion	(8)
Δ*sapRS*	OG1RF *sapRS* deletion	This work
Δ*croRS*	OG1RF *croS* deletion, no expression of *croR*	(17)
Δ*bsrRS*	OG1RF *bsrRS* deletion	This work
Δ*mprF2*	OG1RF *mprF2* deletion	(18)
madE::tn	Tn insertion at site 2392085, Cm^R	(19)
Δ*liaFSR*Δ*sapRS*	OG1RF *liaFSR* and *sapRS* deletion	This work
Δ*liaFSR*Δ*sapRS*Δ*ltaS1*	OG1RF *liaFSR, sapRS* and *ltaS1* deletion	This work
<u>*E. coli*</u>		
Stellar	Host strain of pGCP213, pMSP3535, and pJC005	Lab stock
DH5α	Host strain of empty pMSP3535 plasmid	Lab stock

Plasmid	Description[a]	Reference
pGCP213	Temperature-sensitive plasmid used for allelic exchange; Erm^R: 500 µg/mL *E. coli;* 25 µg/mL *E. faecalis*	(10)
pGCP-*ltaS1*	pGCP213 carrying *ltaS1* deletion insert; Erm^R	This work
pGCP-*ltaS2*	pGCP213 carrying *ltaS2* deletion insert; Erm^R	This work
pGCP-*sapRS*	pGCP213 carrying *sapRS* deletion insert; Erm^R	This work
pJC005.em	CRISPR-Cas12a genome editing plasmid; Erm^R: 200 µg/mL *E. coli;* 50 µg/mL *E. faecalis*	(9)
pJC-*bsrRS*	pjC005 carrying *bsrRS* deletion insert; Erm^R	This work
pMSP3535	Gram-positive, nisin-inducible expression vector; Erm^R: 300 µg/mL *E. coli,* 10 µg/mL *E. faecalis*	(14)
pMSP-*ltaS1*	pMSP3535 carrying the *ltaS1* gene under the control of the nisin-inducible promoter; Erm^R	This work

[a]Fus: fusidic acid; Rif: rifampicin; Erm: erythromycin

Table S2. Antibodies and antibiotics used in this study.

Antigen	Size (kDa)	Primary antibody (concentration; host)	Secondary antibody (concentration; host)	Reference
LTA	10-14	Hycult Clone 55 (1:1000; Mouse)	α-mouse IgG HRP (1:5000; Goat)	(2)
SrtA	27.1	α-SrtA (1:250; Mouse)	α-mouse IgG HRP (1:1000; Goat)	(20)

Antibiotic	Vendor	Reference	Stock concentration
Daptomycin	Thermo Scientific	461375000	10 mg/mL
	Goldbio	D-705	10 mg/mL
Erythromycin	Thermo Scientific	227330050	100 µg/mL
Nisin	Sigma Aldrich	N5764-5G	1 µg/mL
Anhydro-tetracycline	Thermo Scientific	24120625	1 mg/mL

Table S3. Primers used in this study.

Primers	Sequence*	Application
M13 F	5’GTAAAACGACGGCCAGT3’	pGCP213
M13 R	5’CAGGAAACAGCTATGAC3’	screening
yxdJK_del1	5’CTCAAATCCAGCTCGAGCAAATGAAG3’	
yxdJK_del2	5’ATAATAACTGAGGGAAGGCTTCGACAATCATGATTTTAGCC3’	
yxdJK_del3	5’AAATCATGATTGTCGAAGCCTTCCCTCAGTTATTATAATGAAG3’	*sapRS*
yxdJK_del4	5’CAACGCCCGGATCCCTTTATTAAAATC3’	deletion
yxd screen F	5’GCCGATTTTTTTATAAGATTATGC3’	
yxd screen F	5’GTTGATTCTGTCCCAAACTCC3’	
ltaS1_del1	CCGCTTCTCTCTAGATATTTCAAAGAG	
ltaS1_del2	CCCGTTTTTTGATAGTTTTCAAAATAAACGTCC	
ltaS1_del3	GGACGTTTATTTTGAAAACTATCAAAAAACGGG	*ltaS1*
ltaS1_del4	GAGACCACTGCAGAATAGCATCAC	deletion
ltaS1 screen F	CTTGAAGTTTCATTCTGTACACC	
ltaS1 screen R	TCTGGCATCGTTCGTAAAAAC	
ltaS2_del1	GCTATATCTGCAGGAGATGAGAGGC	
ltaS2_del2	AGTCGGATAATTTTGGACTTAAAAAGAGTCCTAATCGGTT	
ltaS2_del3	AACCGATTAGGACTCTTTTTAAGTCCAAAATTATCCGACT	*ltaS2*
ltaS2_del4	CTTTTTTGCTAGATAAGCTTAGCCCATCATCAA	deletion
ltaS2 screen F	TGCGGTGACATGTAAGCAA	
ltaS2 screen R	GCCTGTTGTATTTTCTGTTGATGG	
pJC005-inv-1	CTGGCATCTTTTTATTTAGGGATTTCTCAC	pJC005
pJC005-inv-2	GACATGCAAGCTTGGCACTGGC	cloning
insert-F	GTGAGAAATCCCTAAATAAAAAGATGC	
insert-R	GCCAGTGCCAAGCTTGCATGTC	*bsrRS* deletion
yclRK-screen-F	GGTCAATTCAGGAAACCGC	

yclRK-screen-R	CTCTTGTTTGCGGGACTCC	
ltaS1-comp-F	AATCATGGATCCGAAAGGACGTTTATTTTG	
ltaS1-comp-R	TCTCATTCTAGATCACCTCTCTAAACTATCTTTAG	*ltaS1* complement
pMSP screen F	ACCCGGTTGTAAAACAGGAG	
pMSP screen R	ACGACTCACTATAGGGAGACC	

*Underlined bases correspond to restriction sites included to clone PCR products.

Synthetic inserts

>yclRK-synthetic-insert
GTGAGAAATCCCTAAATAAAAAGATGCCAGTGTGCTGGAATTCGTCCACTAGTCATTCTACA
GTTTATTCTTGACATTGCACTGTCCCCCTGGTATAATAACTAT**AATTTCTACTCTTGTAGAT**ct
cttccgtcatgccgattcctg**AATTTCTACTCTTGTAGAT**aaaaatacggcgctcattgtaaaagccaattcatgaccac
gctgtttgacatgttcatgcagtgaaacacattcctctgttgtaaattcatctgaataaatcgtttttccttcataatgaataaattgcccgttcatt
acaatcgcagaatcaatgccactgtctttcataattggctgaatctcacacaaggtccgtcctgtcgcaattaaaggtaaaatttgattgtctt
tcaaggccgccactgctgcggtaatctcaggagttatttgcgatgttccatctaacaaagttccatctaaatcaaaaaaagtaatagctttc
attttatccataccctaacatcctctctacgcttgttattttaacaaacttttctgaaaagacaatgggttcctttcgttttctttaaaagagcagga
aaaaaagtgatttttgatggttctgtaaaggatttttgaagaaatatagaatcggttggtacaaattcaattttagagtatactacaatagagc
agttttatgaaaagtgaataaggagtgaatgaaagaaaacgtctggtcacaatcagtgccagacgttttttagtttaataacgcatgtaga
gacaaggggcgcacatagggactatcaataatttgtggatcctttgtcagaaatagcaagcgcttgtttctttttgaactgctatttttaattga
ggtaaaatttcttgacgttgtccaatcgttaatgaagaaaagatatcatcaatcactaacgtacttgtttttgaaaacaaaagttgtattatgcg
cattttaatttttcaaaagtcgtaaaagaagtaaaggaacgaccaagtaaggcaggttctaattggaacacctggaaagcttcggctag
aactacttttcgatcccgttctttgatcgtactagcgataaataaattttcttctaatggcaagtagggcaaaaaggtttcactagaagaaag
cctagtaatggttagttggtttgtttggaaaaaatcggccaatgcgagttcttgttctggagcagaataaatgattccataaacttctcgcaaa
gctaacgcgtcgaatgaggatttGACATGCAAGCTTGGCACTGGC

Table S4. DAP sensitivity of *E. faecalis* OG1RF wild-type and mutant strains in BHIc.

Strain	DAP
WT	8
Δ*ltaS1*	0.5
Δ*ltaS2*	8
WT + pMSP3535	8
Δ*ltaS1* + pMSP3535	0.5
Δ*ltaS1* + p-*ltaS1*	4
Δ*bsrRS*	8
Δ*mprF2*	4-8
madE::tn	4
Δ*sapRS*	2
Δ*liaFSR*	0.5
Δ*liaFSR*Δ*sapRS*	0.25
Δ*liaFSR*Δ*sapRS*Δ*ltaS1*	0.125

All values are in µg/mL and were assayed on 3 independent days.
DAP = daptomycin. BHIc = BHI + 50 mg/L $CaCl_2$.

Dataset S1 (separate file). RNA-Seq results comparing daptomycin-treated vs untreated wild-type *E. faecalis* OG1RF. Genes with significant differences in mRNA abundance were determined using a P value and false discovery rate (FDR) cut-off of 0.05. The log_2FC represents the log_2 fold change between biological triplicates of exponentially growing OG1RF when treated for 15 min with 2μg/mL of daptomycin in BHI supplemented with 50mg/L $CaCl_2$. Functional classification of genes was manually curated.

Dataset S2 (separate file). RNA-Seq results comparing treated or untreated OG1RF Δ*liaFSR* and Δ*liaR* strains to treated or untreated wild-type *E. faecalis* OG1RF strain, respectively. Genes with significant differences in mRNA abundance were determined using a P value and false discovery rate (FDR) cut-off of 0.05. The log_2FC represents the log_2 fold change between biological triplicates of exponentially growing OG1RF in BHI supplemented with 50mg/L $CaCl_2$. Functional classification of genes was manually curated.

SI References

1. R. Rashid *et al.*, Comprehensive analysis of phospholipids and glycolipids in the opportunistic pathogen Enterococcus faecalis. *PLoS One* **12**, e0175886 (2017).
2. R. Rashid *et al.*, Depleting Cationic Lipids Involved in Antimicrobial Resistance Drives Adaptive Lipid Remodeling in Enterococcus faecalis. *mBio* **14**, e0307322 (2023).
3. S. Andrews, FastQC: A Quality Control Tool for High Throughput Sequence Data. *Available online at:* http://www.bioinformatics.babraham.ac.uk/projects/fastqc/ (2010).
4. B. Bushnell, BBMap short-read aligner, and other bioinformatics tools. . *University of California, Berkeley, CA.* (2015).
5. H. Li, Aligning sequence reads, clone sequences and assembly contigs with BWA-MEM (arXiv:1303.3997). *arXiv.* https://doi.org/10.48550/arXiv.1303.3997 (2013).
6. M. D. Robinson, D. J. McCarthy, G. K. Smyth, edgeR: a Bioconductor package for differential expression analysis of digital gene expression data. *Bioinformatics* **26**, 139-140 (2010).
7. R. Shrivastava, X. Jiang, S. S. Chng, Outer membrane lipid homeostasis via retrograde phospholipid transport in Escherichia coli. *Mol Microbiol* **106**, 395-408 (2017).
8. Z. J. Nair *et al.*, An essential protease, FtsH, influences daptomycin resistance acquisition in Enterococcus faecalis. *Mol Microbiol* **121**, 1021-1038 (2024).
9. M. J. Chua, J. Collins, Rapid, Efficient, and Cost-Effective Gene Editing of Enterococcus faecium with CRISPR-Cas12a. *Microbiol Spectr* **10**, e0242721 (2022).

10. H. V. Nielsen *et al.*, The metal ion-dependent adhesion site motif of the Enterococcus faecalis EbpA pilin mediates pilus function in catheter-associated urinary tract infection. *mBio* **3**, e00177-00112 (2012).
11. J. F. Watson, J. García-Nafría, In vivo DNA assembly using common laboratory bacteria: A re-emerging tool to simplify molecular cloning. *J Biol Chem* **294**, 15271-15281 (2019).
12. S. Arroyo-Urea, J. F. Watson, J. García-Nafría, Molecular Cloning Using In Vivo DNA Assembly. *Methods Mol Biol* **2633**, 33-44 (2023).
13. J. T. Robinson *et al.*, Integrative genomics viewer. *Nat Biotechnol* **29**, 24-26 (2011).
14. E. M. Bryan, T. Bae, M. Kleerebezem, G. M. Dunny, Improved vectors for nisin-controlled expression in gram-positive bacteria. *Plasmid* **44**, 183-190 (2000).
15. A. Bourgogne *et al.*, Large scale variation in Enterococcus faecalis illustrated by the genome analysis of strain OG1RF. *Genome Biol* **9**, R110 (2008).
16. G. M. Dunny, B. L. Brown, D. B. Clewell, Induced cell aggregation and mating in Streptococcus faecalis: evidence for a bacterial sex pheromone. *Proc Natl Acad Sci U S A* **75**, 3479-3483 (1978).
17. S. Mikhaleva *et al.*, Adaptation of Enterococcus faecalis to intestinal mucus revealed by a human colonic organoid model. *mSystems* 10.1128/msystems.01304-25, e0130425 (2026).
18. K. Kandaswamy *et al.*, Focal targeting by human β-defensin 2 disrupts localized virulence factor assembly sites in Enterococcus faecalis. *Proc Natl Acad Sci U S A* **110**, 20230-20235 (2013).
19. C. J. Kristich *et al.*, Development and use of an efficient system for random mariner transposon mutagenesis to identify novel genetic determinants of biofilm formation in the core Enterococcus faecalis genome. *Appl Environ Microbiol* **74**, 3377-3386 (2008).
20. C. Colomer-Winter *et al.*, The HtrA chaperone monitors sortase-assembled pilus biogenesis in Enterococcus faecalis. *PLoS Genet* **20**, e1011071 (2024).
